# The complete mitochondrial genome of the toad species *Oreolalax xiangchengensis* (Anura: Megophryidae) and phylogenetic analyses

**DOI:** 10.1080/23802359.2018.1535842

**Published:** 2018-10-26

**Authors:** Shize Li, Xinyu Gao, Gang Wei, Bin Wang, Ning Xu

**Affiliations:** a Department of Food Science and Engineering, Maotai University, Zunyi, China;; b Chengdu Institute of Biology Chinese Academy of Sciences, Chengdu, China

**Keywords:** *Oreolalax xiangchengensis*, complete mitochondrial genome, phylogenetic analyses

## Abstract

*Oreolalax xiangchengensis* is an alpine toad species of Megophryidae. We determined its complete mitogenome. The mitogenome has a length of 17,110 bp, containing 13 protein-coding genes, 2 rRNA genes, 23 tRNA genes, and a control region. It has a tandem duplication of *tRNA-Met* gene and a translocation of *tRNA-Trp* gene compared with typical vertebrate mitogenomes. Phylogenetic analyses based on the mitogenome sequences of 18 Megophryidae species were conducted to access phylogenetic assignments of *O. xiangchengensis*.

The toad genus *Oreolalax* belongs to Megophryidae, which is distributed in the southwestern China and northern Vietnam (Frost 2018). However, phylogenetic relationships of the genus were still in doubt (Wei et al. 2009). *Oreolalax xiangchengensis* is distributed in the eastern edge of Tibetan Plateau at the most west of the distribution range of *Oreolalax*, and it is an endemic species in China. The complete mitogenome would promote phylogenetic study of the species.

Here, we reported the complete mitogenome of *O. xiangchengensis*. One female *O. xiangchengensis* (topotypic specimen CIB20130642) was collected from Xiangcheng County, Sichuan Province, China, and was preserved in 95% ethanol. The specimen was preserved in Chengdu Institute of Biology, Chinese Academy of Sciences. Total DNA was extracted from the tissue using the DNeasy Tissue Kit (QIAGEN). The mitogenome was deposited in GenBank with the accession number MH727696. It has a length of 17,110 bp, containing 13 protein-coding genes, 2 rRNA genes, 23 tRNA genes, and a control region. The base composition of A + T (62.2%) was higher than G + C (37.8%), most common with vertebrates (Boore 1999). It has a tandem duplication of *tRNA-Met* gene and a translocation of *tRNA-Trp* gene from the ‘WANCY’ region to the location between *tRNA-Pro* gene and control region.

We used MEGA7 to construct the phylogenetic tree using the maximum-likelihood method (Figure 1) based on the mitogenome sequences of *O. xiangchengensis* of other 17 Megophryidae species and two outgroups according to the previous study (Frost et al. 2006). The results support that the genus *Oreolalax* is a monophyly and *O. xiangchengensis* are deeply clustered into this genus.

**Figure 1.**
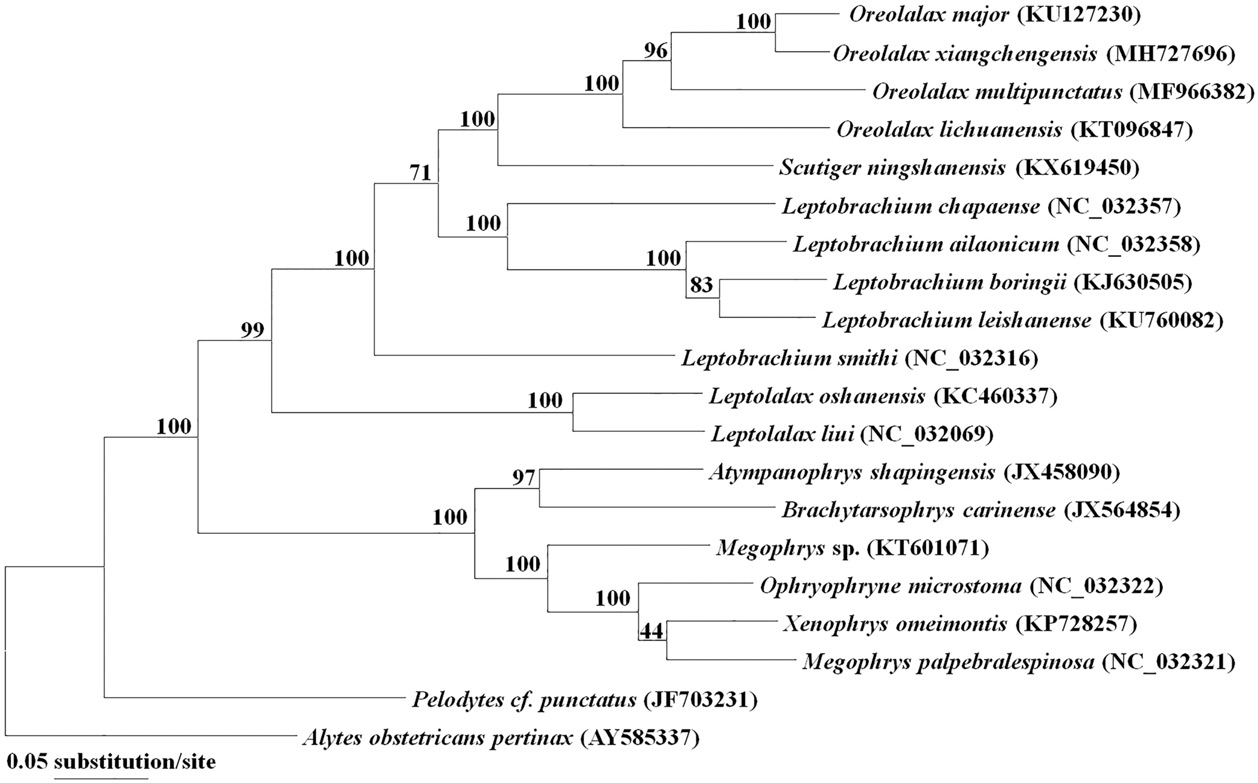
Maximum likelihood tree based on mitogenome sequences of 18 Megophryidae species and two outgroups. Numbers nodes are bootstrap supports.
